# Three-Dimensional Oral Mucosal Equivalents as Models for Transmucosal Drug Permeation Studies

**DOI:** 10.3390/pharmaceutics15051513

**Published:** 2023-05-17

**Authors:** Azra Riaz, Sanna Gidvall, Zdenka Prgomet, Aura Rocio Hernandez, Tautgirdas Ruzgas, Emelie J. Nilsson, Julia Davies, Sabrina Valetti

**Affiliations:** 1 Biomedical Science, Faculty of Health and Society, Malmö University, 205 06 Malmö, Sweden; 2 Biofilms–Research Center for Biointerfaces (BRCB), Malmö University, 205 06 Malmö, Sweden; 3 Section for Oral Biology and Pathology, Faculty of Odontology, Malmö University, 205 06 Malmö, Sweden

**Keywords:** oral transmucosal delivery, oral mucosal equivalents, drug permeation, 3R principles, 3D in vitro models

## Abstract

Oral transmucosal administration, where drugs are absorbed directly through the non-keratinized, lining mucosa of the mouth, represents a solution to drug delivery with several advantages. Oral mucosal equivalents (OME) developed as 3D in vitro models are of great interest since they express the correct cell differentiation and tissue architecture, simulating the in vivo conditions better than monolayer cultures or animal tissues. The aim of this work was to develop OME to be used as a membrane for drug permeation studies. We developed both full-thickness (i.e., connective plus epithelial tissue) and split-thickness (i.e., only epithelial tissue) OME using non-tumor-derived human keratinocytes OKF6 TERT-2 obtained from the floor of the mouth. All the OME developed here presented similar transepithelial electrical resistance (TEER) values, comparable to the commercial EpiOral™. Using eletriptan hydrobromide as a model drug, we found that the full-thickness OME had similar drug flux to EpiOral™ (28.8 vs. 29.6 µg/cm^2^/h), suggesting that the model had the same permeation barrier properties. Furthermore, full-thickness OME showed an increase in ceramide content together with a decrease in phospholipids in comparison to the monolayer culture, indicating that lipid differentiation occurred due to the tissue-engineering protocols. The split-thickness mucosal model resulted in 4–5 cell layers with basal cells still undergoing mitosis. The optimum period at the air–liquid interface for this model was twenty-one days; after longer times, signs of apoptosis appeared. Following the 3R principles, we found that the addition of Ca^2+^, retinoic acid, linoleic acid, epidermal growth factor and bovine pituitary extract was important but not sufficient to fully replace the fetal bovine serum. Finally, the OME models presented here offer a longer shelf-life than the pre-existing models, which paves the way for the further investigation of broader pharmaceutical applications (i.e., long-term drug exposure, effect on the keratinocytes’ differentiation and inflammatory conditions, etc.).

## 1. Introduction

### 1.1. Interest in Research in Mucosal Tissue

Mucosal tissue is the protective layer that covers the surfaces of our internal organs, providing the primary barrier against viruses and bacteria. Mucosal disorders affect over half the world’s population, from infants to the elderly, and place an enormous burden on healthcare budgets. Recently, mucosa has been of particular interest as the primary route of SARS-CoV-2 infection [1]. Oral transmucosal administration, where drugs are absorbed directly through the non-keratinized, lining mucosa of the mouth (e.g., sublingual and buccal), represents a solution to drug delivery with several advantages. Firstly, it is a non-invasive, cost-effective and patient-friendly route for drug administration. Rapid drug absorption via the superior vena cava also allows fast therapeutic onset, avoiding both gastric and first-pass metabolism [2,3].

### 1.2. Tissue Architecture and Non-Keratinized Epithelium

Mucosae are self-renewing tissues where cells in the deepest layers undergo cell division followed by terminal differentiation as they migrate to the surface. Mucosae are generally composed of a superficial epithelial tissue that can be keratinized or non-keratinized, and an underlying fibrous connective tissue. Full differentiation in keratinized epithelia (e.g., masticatory mucosa or skin) leads to the production of the stratum corneum, where the flat, hexagonal cells are surrounded by an external lipid matrix that replaces the plasma membrane. The intercellular lipid matrix in the stratum corneum shows a unique lamellar arrangement that is crucial for the permeation barrier. In non-keratinized epithelia the accumulation of lipids is less evident, and the presence of lamellar structures is far less marked [4]. Thus, the lining mucosa of the oral cavity represents a weaker barrier than the keratinized counterpart, making this the target site for oral transmucosal drug delivery.

### 1.3. Status and Unmet Need of Mucosal Equivalents for Pharmaceutical Applications

To develop new therapeutic opportunities and gain novel insights, models of human mucosa are currently used in experimental research. Recently, tissue engineering has permitted the reconstruction of human oral mucosa as an alternative to animal models (e.g., porcine mucosae) for several applications, including the investigation of fungal and bacterial infections [5,6]. Oral mucosal equivalents (OME) developed as 3D in vitro models are of great interest since they express the correct cell differentiation and tissue architecture, simulating the in vivo conditions better than monolayer cultures or animal tissues (see Moharamzadeh et al., 2007 for an extensive review on the subject) [7]. In fact, they express most of the structural and histological characteristics of normal tissue, such as the development of a polarized cell phenotype, cell–cell contacts, stratification, differentiation and the expression of adhesion molecules, chemokines and cytokines [8,9]. OME can be developed as full- or split-thickness models. The full-thickness model includes a connective tissue layer (lamina propria) covered by an epithelium containing epithelial cells [10]. The lamina propria is a 3D scaffold (usually made of collagen) infiltrated by fibroblasts producing an extracellular matrix, while the upper layer is a stratified squamous epithelium including densely packed keratinocytes that undergo differentiation as they migrate to the surface. The split-thickness model comprises only the stratified squamous epithelium, where keratinocytes are directly lying on permeable cell culture membranes at the air–liquid interface [7]. Since the split-thickness model represents the epithelium barrier, it is appropriate for permeation studies, being a shorter and cheaper tissue-engineering protocol than the full-thickness one. The MatTek Corporation (Ashland, MA) offers commercial full- or split-thickness models of non-keratinized OME (i.e., EpiOral™) that form a multilayer and stratified epithelium that exhibits in-vivo-like morphological and growth characteristics [11]. EpiOral™ has been used as barrier model to study drug permeation in several studies [3] since it presents a similar lipid composition to the human counterpart. However, the primary cells used in this model have a limited number of passages and do not survive for a long time in vitro. Since the full-thickness OME take 3–4 weeks to develop into the differentiated, stratified mucosal tissue, this would limit the time available for experimentation with the model as the cells reach their passage limit. Dongari-Bagtzoglou et al. have developed an OME using OKF6 TERT-2 keratinocytes instead of primary cells [6]. Thanks to immortalization via the forced expression of telomerase 2, OKF6 TERT-2 retain their normal growth and differentiation characteristics without developing a cancerogenic phenotype [12,13]. The OME model constructed with OKF6 TERT-2 simulates the buccal mucosa in terms of epithelial cell stratification and differentiation and the expression of adhesion molecules (e.g., E-Cadherin) and the proliferation marker Ki-67 [6]. This model has also been assessed as a useful tool in the study of host–pathogen interactions in oral candidiasis [6,14]. However, to the best of our knowledge, a split-thickness model has not been developed yet and no investigations of the barrier model have been performed for drug permeation studies. Furthermore, in the last few years, tissue-engineering protocols have been modified to implement the European Community directive 86/609/EE, the so-called 3R principles that aim to reduce, refine, or replace the use of laboratory animals [15].

In this context, the aim of our study was to develop full- and split-thickness OME using the human keratinocyte OKF6 TERT-2 cell line and to assess the permeation barrier properties in relation to the commercial model (i.e., EpiOral™). Using the 3R principles, we investigated the effect of replacing animal-derived compounds (i.e., fetal bovine serum) on the tissue-engineering protocol. Finally, we evaluated the lipid composition of the full-thickness OME to understand the barrier properties in more depth.

## 2. Materials and Methods

### 2.1. Materials

Eletriptan hydrobromide (EB, CAS No. 177834-92-3) was purchased from Jiangsu Zenji Pharmaceuticals LTD. (Huaian, China). Acrodisc syringe filters (PVDF and PTFE, 13 mm with 0.2 µm pores), HPTLC silica plates 60 f254, phosphate-buffered saline (PBS, 137 mM NaCl, 2.7 KCl and 10 mM phosphate buffer pH 7.4, E404-100 TABS), methanol, acetonitrile, chloroform, hexane, dichloromethane and formic acid (>99%) were all purchased from VWR Chemicals (Stockholm, Sweden). All solvents used were HPLC-grade. The following standard lipids used as a reference for HPTLC were obtained from Avanti^®^ Polar Lipids (Merck, Stockholm, Sweden): cholesterol 3-sulphate, cholesteryl oleate, l-α-phosphatidylinositol (bovine liver, PI), 1,2-dioleoyl-sn-glycero-3-phosphocholine (≥99%, DOPC) and ceramide NS (N-lignoceroyl-D-erythro-sphingosine). The remaining standard lipids, from Sigma-Aldrich (Merck, Stockholm, Sweden), were l-α-phosphatidylethanolamine, dioleoyl (≥98% (TLC), DOPE), galactocerebrosides (from bovine brain, ≥97% (TLC), ceramide β-D-galactoside), sphingomyelin (from bovine brain ≥ 97.0%), cholesterol, oleic acid (≥99%), glyceryl trioleate (≥99%). For the ceramide (Cer) subclassification, we used the nomenclature based on the structure of the individual Cer chains first introduced by Motta et al. [16] and broadly used in skin research [17]. Cupric sulphate of technical grade and phosphoric acid were purchased from VWR Chemicals (Stockholm, Sweden). The medium and supplements for cell culture were purchased from Gibco, Fisher Scientific G10TF AB, Gothenburg, Sweden.

### 2.2. Composition of Cell Culture Media and Buffers

Keratinocyte growth medium (KGM) was prepared by supplementing keratinocyte serum-free medium (KSFM) with 1 IU penicillin/mL, 1 μg/mL streptomycin, 0.3 mM CaCl_2_, 25 μg/mL bovine pituitary extract and 0.2 ng/mL human recombinant epidermal growth factor [13,18]. High-density medium (HDM) was prepared by mixing KGM with an equal volume of a mixture of Dulbecco’s modified Eagle medium (DMEM) and nutrient mixture Hams F12 mixed in a ratio of 1:1 (*v*/*v*), supplemented with bovine pituitary extract 25 μg/mL, human recombinant epidermal growth factor 0.2 ng/mL, penicillin 1 IU/mL, streptomycin 1 μg/mL and CaCl_2_ 0.3 mM.

Culture medium for the NIH 3T3 fibroblasts was DMEM (with 4.5 g/L glucose, L-glutamine and sodium pyruvate) supplemented with 10% FBS (Sigma) and penicillin 1 IU/mL, streptomycin 1 µg/mL [6].

Reconstitution buffer 10× for the preparation of collagen gels was prepared by dissolving 22 mg/mL sodium bicarbonate and 20 mM HEPES in 0.062 N NaOH [6].

Air–liquid interface (ALI) medium for full- and split-thickness models was prepared by combining DMEM (with 4.5 mg/mL glucose) and Ham’s F-12 in a 3:1 ratio, supplemented with 5 μg/mL insulin, 0.4 μg/mL hydrocortisone, 2 × 10^−11^ M 3,3′ 5 triiodo-L-thyronine, 1.8 × 10^−4^ M adenine, 5 μg/mL transferrin, 10^−10^ M cholera toxin, 2 mM L-glutamine, 5% FBS, 1 IU/mL penicillin–streptomycin and fungizone 0.5 μg/mL.

### 2.3. Cell Culture

The immortalized normal human oral keratinocyte OKF6 TERT-2 cell line was obtained from Dr. James Rheinwald (Department of Dermatology, Brigham and Women’s Hospital, Boston, MA, USA) at passage 33, and monolayer cultures were expanded through passage 33 to 40 at 37 °C and 5% CO_2_ in KGM. Cell seeding density was 2.8 × 10^4^ cells/mL, the medium was replaced every two days and the cells were divided when reaching 60–70% of confluence. Cells were detached by incubation with trypsin supplemented with 0.53 mM EDTA, followed by two centrifugation steps (100 x g, room temperature, 10 min). To achieve the differentiation of keratinocytes into their characteristic mature, large and octagonal form, the KGM was replaced with HDM when cells achieved around 50–60% confluence. Cells were treated with HDM for 2–3 days to obtain a confluent, differentiated monolayer of OKF6 TERT-2 cells, as described previously [13]

The NIH 3T3 fibroblast cell line was initiated at passage 14 and expanded through passages 15 to 27. The cells were maintained in fibroblast growth medium at 37 °C and 5% CO_2_ and passaged twice weekly. Seeding density was 1.25 × 10^5^ cells/mL.

### 2.4. Full-Thickness Tissue-Enginereed Oral Mucosa

**Figure 1:**
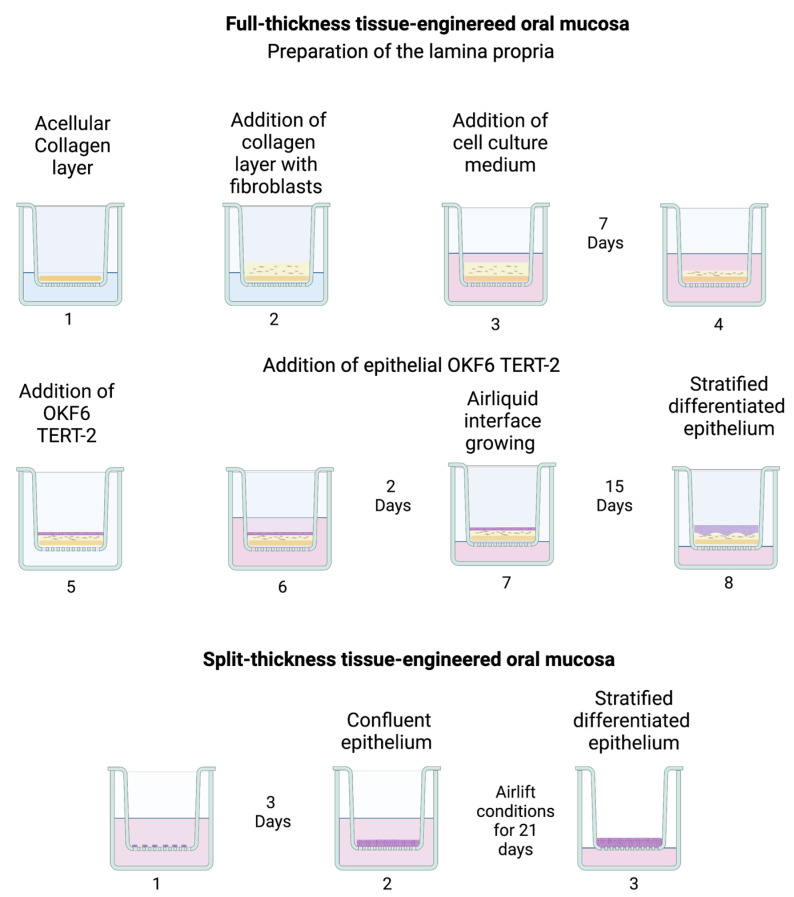
Outline of the tissue-engineering protocol with numbered steps.

Full-thickness tissue-engineered oral mucosa was cultured as already described previously, with minor modifications [6]. This consisted of two steps: the preparation of acellular and cellular collagen gels (i.e., lamina propria) followed by the addition of epithelial OKF6 TERT-2 cells (i.e., epithelium). A schematic of the tissue engineered protocols is presented in Figure 1.

#### 2.4.1. Preparation of the Lamina Propria

Lamina propria needed for the full-thickness model was constructed by growing fibroblasts embedded in collagen on a layer of acellular collagen in a 12-well plate insert (Sarstedt, pore size 0.4 μm, PET). Acellular collagen for four inserts was prepared by mixing 0.1 mL of 10× DMEM, 0.1 mL of reconstitution buffer 10×, 0.45 mL of FBS, 0.005 mL of 2 mM L-glutamine and 0.6 mL of 8.9 mg/mL rat tail collagen type I on ice. The final collagen concentration was 4.25 mg/mL. Using a pre-cooled pipette, 0.2 mL of the mixture was dispensed into each insert and allowed to solidify for 30 min. This was followed by placing a layer of collagen-embedded fibroblasts on top of the acellular collagen layer. Fibroblast-embedded collagen (cellular gels) was prepared by mixing 0.2 mL of 10× DMEM, 0.2 mL of reconstitution buffer 10×, 0.5 mL of FBS, 0.03 mL of 2mM L-glutamine, 0.9 mL of 8.9 mg/mL collagen and 0.2 mL of 4 × 10^6^ cells/mL NIH 3T3 cell suspension. The final collagen concentration was 3.95 mg/mL. Then, 0.4 mL of fibroblast-embedded collagen gel was poured into each insert on top of the acellular gel and left for 1 h at room temperature, followed by 1 h at 37 °C and 5% CO_2_. Next, 1.5 mL of DMEM with 10% FBS was added both to the apical and basolateral sides. The cellular gels were incubated for 7 days to allow gel contraction.

#### 2.4.2. Addition of Epithelial OKF6 TERT-2

Culture medium was aspirated from the inserts and wells, followed by the addition of 0.6 mL fresh medium to the basolateral side of the wells. This was followed by the addition of 0.1 mL cell suspension containing 5 × 10^5^ OKF6 TERT-2 cells to the middle of each insert. The plate was incubated for 2 h at 37 °C and 5% CO_2_ so that cells adhered to the cellular gels. Then, 1 mL of KGM was added to the apical side and 1.5 mL DMEM-10% FBS growth medium was added to the basolateral side. The plate was incubated at 37 °C and 5% CO_2_. The KGM was replaced the next day. After 2 days, the medium was aspirated from the inserts and 0.6 mL of ALI medium was added to the basolateral side of the well. The cultures were incubated at 37 °C and 5% CO_2_ and the media replaced with of 0.3 mL fresh ALI medium every other day for a total of 15 days.

### 2.5. Split-Thickness Tissue-Engineered Oral Mucosa

Split-thickness OME were cultured using Costar Transwell inserts with polyethylene membranes (PE), Millipore inserts with polycarbonate membranes (PC) or Sarstedt inserts with polyethylene tetraphthalate (PET) membranes. OKF6 TERT-2 cells were seeded at a density of 1 × 10^6^ cells (p 36–37) in 0.1 mL of KGM directly onto the insert membranes and cultured under submerged conditions for 3 days until confluence was reached. After 3 days, the culture medium from both the apical and basolateral sides of the wells was removed and 0.6 mL ALI medium was added to the basolateral side and the cells cultured for 21 days at ALI with the replacement of 0.3 mL fresh ALI medium every other day.

The influence of the Ca^2+^ concentration in the ALI medium was tested by adding an additional 1 mM CaCl_2_. Moreover, the composition of the ALI medium was improved according to the 3R principles. In particular, FBS was replaced with 10 μM retinoic acid, 5 μg/mL linoleic acid and 0.2 ng/mL EGF. The influence of an additional 25 μg/mL BPE was also tested. Specifications of the composition of the air–liquid interface medium (ALI) for all tissue-engineered mucosa models are given in Table S1, Supplementary Materials.

### 2.6. Histology of Tissue-Enginereed Oral Mucosa

Full- and split-thickness mucosae was fixed in formalin, dehydrated and embedded in paraffin and sectioned at 3 µm. All sections were stained with Hematoxylin–Eosin (H&E) staining using Tissue-Tek Prizma Stainer from Sakura.

### 2.7. Drug Permeation Studies across Tissue-Enginereed Oral Mucosa

The permeation of eletriptan hydrobromide (EB) was evaluated across the in-house-developed tissue-engineered oral mucosa, commercial tissue-engineered oral mucosa EpiOral™ ORL-200 (MatTek, Ashland, MA, USA) and collagen gels (acellular plus cellular). Prewarmed KGM or DMEM with 10% FBS was added to the in-house-developed full-thickness mucosa and acellular collagen gels, respectively. In accordance with the supplier’s instructions, EpiOral™ ORL-200 was incubated with prewarmed EpiOral assay medium and placed at 37 °C, 5% CO_2_ for a 1 h equilibration. The tissues were placed at room temperature for 30 min and TEER was measured. The drug permeation experiment was initiated by replacing the apical solutions with 3 mg/mL EB at pH 6. A sample of the apical solution was saved for the estimation of the initial drug concentration by HPLC analysis. For each time point, the inserts containing the tissues were moved to new wells containing fresh assay medium (i.e., on the basolateral side). The tissues were moved from well to well at the appropriate time points and the basolateral fluid was collected from each well. The basolateral samples were vortexed for 1 min with an equal volume of methanol and evaluated with HPLC (Section 2.8). At the end of the experiments, the apical solution was taken for mass balance analysis and to ensure that the apical solution concentration had remained constant throughout the experiment.

### 2.8. Drug Quantification in Permeation Experiments

The samples collected from the permeation experiments were analyzed with HPLC-UV (Agilent 1100 Series, Agilent Technologies, Mulgrave, Australia) using a method previously reported [19]. Briefly, a SVEA HPLC C_18_ column (4.6 × 100 mm, 3.5 μm, Nanologica, Södertälje, Sweden) with a Phenomenex SecurityGuard precolumn with cartridges C_18_ 4 × 3.0 mm ID (AJO-4287 Phenomenex, Værløse, Denmark) was used. The flow rate of the mobile phases was 0.8 mL/min. Mobile phase A consisted of 0.1% formic acid in water, while mobile phase B consisted of 0.1% formic acid in water:acetonitrile (5:95 *v*/*v*). The solvents and mobile phases were degassed before use. The gradient steps were as follows: 0–0 min 15% of B, 0–6 min 95% of B and 7–9 min 15% of B, flow rate of 0.8 mL/min and injection volume of 100 μL. The detection wavelength was λ = 274 nm. The retention time for EB was 4.5 min. Before HPLC analysis, apical and basolateral samples were diluted 1:2 with MeOH, vortexed for 1 min and then filtered with PVDF or PTFE 0.22 μm pore (Millipore, Burlington, MA, USA) filters. A calibration curve for the analysis was performed by preparing standard solutions ranging from 7.8 to 500 μg/mL, dissolved in the same medium used during the respective experiment. LOD and LOQ values (0.872 and 2.643 mg/mL) were calculated with the standard deviation of the response and the slope according to the International Conference of Harmonization (ICH) [20].

### 2.9. Transepithelial Electrical Resistance (TEER)

Transepithelial electrical resistance (TEER) was measured with two chop-stick electrodes composed of glassy carbon rods (1 mm in diameter), which were wound with a 0.2-mm-diameter platinum wire to ensure a large surface area. The electrodes were connected to a potentiostat (IVIUM Technologies, Eindhoven, The Netherlands). To limit the electrical current through the cell layers during TEER measurements, 1 kΩ resistance was coupled between the electrodes and the potentiostat. Electrodes were sterilized by immersion in 70% ethanol for 1 h and left to dry under sterile conditions prior to the measurements. Inserts with or without a cultured OME layer were placed in a cell culture plate filled with 0.5 mL of PBS (pH 6.8) in both the apical and basolateral compartments to ensure electrolyte solution above and below the insert membrane. The chop-stick electrodes were then inserted into the PBS above and below the insert and the impedance spectrum between 1 Hz and 1 MHz was recorded. The applied AC voltage had 10 mV amplitude. To calculate TEER values, only total impedance values at 12.5 kHz were used. The TEER value (in Ω) of OME was equal to the difference in the impedance values measured for inserts with OME and an empty insert/membrane (i.e., blank impedance). The final reported TEER values (Ω·cm^2^) were obtained by multiplying the value in Ω by the surface area of the insert membrane. The construction of the electrodes for TEER measurements is presented in the Supplementary Materials. For all pair-wise comparisons of means, Student’s independent *t*-test was performed with a significance level of *p* < 0.05.

### 2.10. TEER Measurements for Evaluation of 3D Growth, Shelf-Life and Tissue Integrity

To measure TEER during air–liquid cell culture, fresh ALI medium at 37 °C was added to the well outside the insert 30 min prior to the measurements and then cultures were equilibrated at room temperature. Shelf-life measurements were taken on untreated mucosae to understand the stability of the model after full differentiation.

For the commercial tissue-engineered oral mucosa EpiOral™, this stability was studied after receiving the tissue and handling it as recommended by the supplier.

Before each permeation experiment, permeation buffer (i.e., PBS at pH 6.8) at room temperature was placed on both the apical and basolateral sides. To check the tissue integrity after the permeation studies, TEER measurements were repeated at the end of the experiments and after 24 h equilibration. For this purpose, at the end of the permeation experiment, the apical solution and basolateral medium were removed, fresh medium was added to the basolateral side, and the tissue was kept in the incubator for an additional 24 h.

### 2.11. Lipid Extraction from Cell Monolayer and Tissue-Enginereed Oral Mucosa

OKF6 TERT2 was cultured in T175 flasks (p 37) at a seeding density of 5 × 10^6^ in 35 mL keratinocyte growth medium. The medium was changed every 2 days till 60% confluence was reached. To obtain a differentiated monolayer, the cells were incubated to 60% confluence in high-density medium (HDM) for 3–4 days until they appeared large and octagonal.

The monolayer and full-thickness tissue-engineered oral mucosa were incubated with trypsin, washed with PBS and then suspended in methanol and dried in a vacuum desiccator for 48 h. Lipids were extracted from the mucosal tissue using a modified Bligh and Dyer method [21]. The solvents used for lipid extraction were chloroform and methanol in three different mixtures (1:2, 1:1 and 2:1); between each solvent step, the extraction was filtered using a pre-burnt glass filter and the solvents were evaporated using a rotavapor. After two sets of extraction at 37 °C, on the same tissue, the combined organic phase was washed with 10 mM KCl and left to separate. The organic phase was collected, and the aqueous phase was washed with chloroform and left to separate again. Both organic phases were collected, i.e., the lipid extracts were combined and filtered, and the solvent was evaporated. The extracted lipids were quantified by weight.

### 2.12. High-Performance Thin-Layer Chromatography (HPTLC)

**Figure 2:**
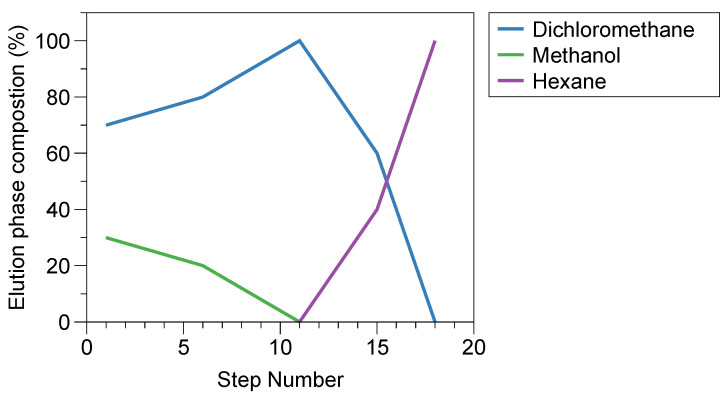
Schematic representation of the 18-step gradient used for HPTLC elution.

The lipid composition of the tissue-engineered oral mucosa was evaluated using high-performance thin-layer chromatography (HPTLC). The HPTLC method was adjusted from Diaz-del Consuelo et al. [22] to be performed without an Automatic Multiple Development (AMD) apparatus. Pre-coated silica plates HPTLC 60 f254 (10 × 20 cm, 200 µm silica thickness, 5–7 µm particle size and 60 Å pore size) were prewashed with MeOH and activated for 60 min at 110 °C and then left to cool before the lipids were applied. Lipids (10–100 µg) were applied as 1-cm-long bands, 2 cm from the bottom edge of the plate and at a distance of 1 cm from each other. Reference standard lipids were applied to identify and quantify the lipid classes on each HPTLC plate. Prior to application, epithelial lipids and standards were dissolved in a 1:1 mixture of chloroform:methanol, with concentrations ranging from 1 to 5 mg/mL depending on the sample. Phospholipids were added with twice the mass compared to the other lipids, to make them easier to detect. The elution was composed of an 18-step gradient program using dichloromethane, methanol and hexane (Figure 2).

For each step, the solvents were allowed to migrate 5 mm further than the previous step; by doing this, the lipids were separated over the plate, based on polarity. Between each solvent step, the plates were dried with acidified N_2_ (obtained by bubbling it through a 0.5% acetic acid solution). The plates were developed by spraying a solution of 10% (*w*/*v*) cupric sulphate in 8% phosphoric acid. After 10 min of drying, the plates were incubated at 155 °C for 10 min. A chromatogram was generated from the HPTLC plates, where each lipid spot generated one peak in the chromatogram. The total area of all peaks in each mixture was summarized and then the area of each individual peak divided by the total; therefore, the value in this graph is expressed as the % of the total amount of added lipids. Chromatograms were evaluated using a Bio-Rad Imaging Densitometer and ImageJ (version 2.0.0-rc-69/1.52p 2018).

### 2.13. Data Analysis for Permeation Experiment

We determined the permeability of EB under steady state conditions, which, in general, are fulfilled under infinite dose sink conditions. This may be expressed according to the generalized Fick’s first law of diffusion:

(1) $$J=-\frac{D\left(x\right)}{RT}c\left(x\right)\frac{d\left(µ\right)}{dx}$$

where J is the flux (mg cm^−2^ h^−1^), *D*(*x*) (cm^2^ h^−1^) is the diffusion coefficient at position *x* (cm), *c*(*x*) (mg cm^−3^) is the concentration of the diffusing molecule at position *x* (cm) and d(µ)/dx (J mol^−1^ cm^−1^) is the derivative of the chemical potential of the diffusing molecule as function of the position *x*. It is evident from Equation (1) that if the chemical potential of the drug is kept at similar values (irrespective of formulation type), the flux from the various formulations can be compared in an unbiased manner. The cumulative amount of drug permeated per unit area (mg/cm^2^) was plotted against the collection time (min). The slope of this graph in the most linear region was considered as the flux (*J*), as reported previously [23]. The results were presented as mean ± standard deviation. The Dixon test was used to detect outliers.

## 3. Results and Discussion

### 3.1. Development and Characterization of Full- and Split-Thickness Oral Mucosa Models

**Figure 3:**
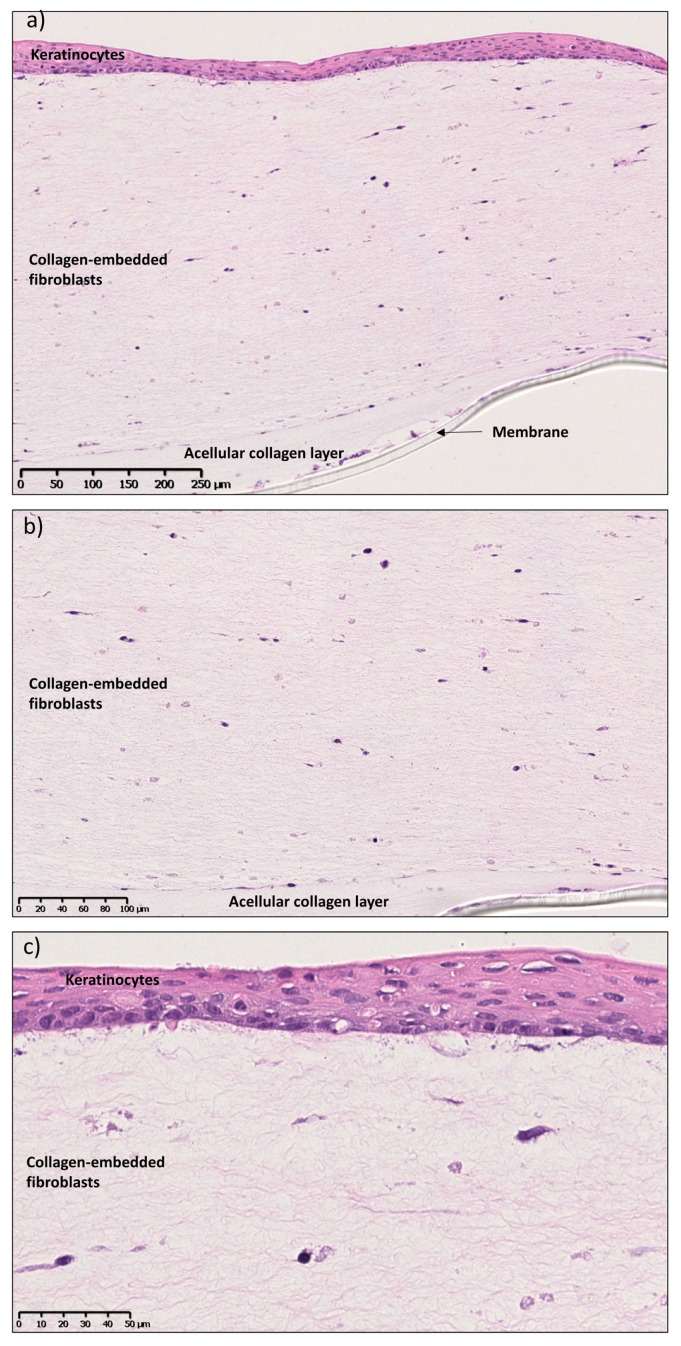
Histological images after H&E staining of full-thickness mucosa after 15 days at (**a**) ×10, (**b**) ×20 and (**c**) ×40 magnification. Scale bars are 250, 100 and 50 µm for (**a**,**b**,**c**) respectively.

In this study, the in vitro 3D oral mucosal equivalents (OME) were developed as full- and split-thickness models. The epithelial component was composed of a non-keratinized, stratified, squamous epithelium resting on the connective tissue component comprising collagen-embedded fibroblasts. OKF6 TERT-2 cells were chosen as keratinocytes to build the epithelium layer since they are very similar to the primary cells of the buccal mucosa in terms of morphology, as opposed to the highly modified carcinoma cell line TR 146 [6], and they serve as a good model to study the potential cytotoxic effects of drugs and particles. The full-thickness tissue-engineered mucosa was cultured as previously shown by Dongari-Bagtzoglou et al. [6], with minor modifications (Figure 1). Figure 3a reveals a well-developed full-thickness mucosa with layers of keratinocytes resting on a layer of lamina propria composed of collagen-embedded fibroblasts and an acellular collagen layer at the bottom. The lamina propria consisted of collagen-secreting fibroblasts, as seen by the high concentration of connective tissue compared to acellular collagen (Figure 3b). The epithelial component was stratified (7–8 layers) with a basal layer of keratinocytes with characteristic rounded nuclei and a surface layer of cells with flattened or rounded nuclei (Figure 3c). The degree of stratification and differentiation was similar to that in the commercial OME model EpiOraI^TM^ (Mattek, Ashland, MA, USA) (see Supplementary Materials, Figure S1).

**Figure 4:**
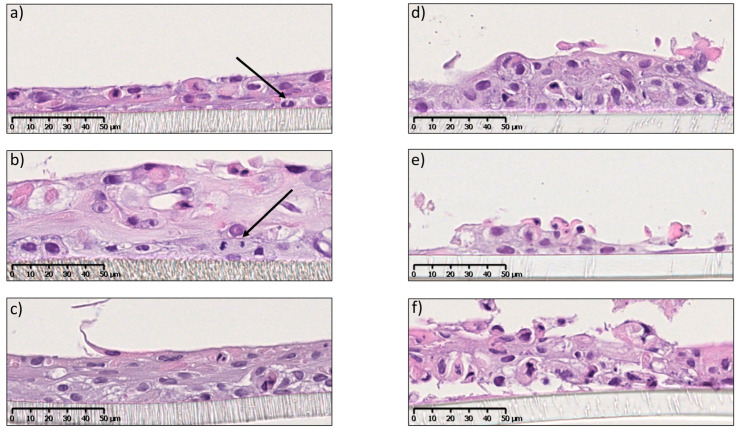
An overview of H&E-stained split-thickness mucosa grown on Sarstedt polyethylene membrane and supplemented with Ca^2+^ after (**a**) 14, (**b**) 18 or (**c**) 21 days in ALI and without supplemented Ca^2+^ after (**d**,**e**) 14 or (**f**) 28 days in ALI. Arrows point to cells undergoing mitosis. Scale bars are 50 µm.

The split-thickness mucosa consisted of a non-keratinized, stratified epithelial layer resting on a permeable and inert membrane. To the best of our knowledge, a tissue-engineering protocol for split-thickness mucosa using OKF6-TERT-2 (Figure 1) has not been shown before; therefore, we chose to optimize the cell medium and number of days at the air–liquid interface (ALI). Figure 4a shows the development of the stratified epithelium consisting of 3–4 layers of keratinocytes, with cells undergoing mitosis after 14 days at ALI. After 18 days at ALI, the epithelium was further stratified into 4–5 layers, with basal cells still undergoing mitosis (Figure 4b). After 21 days, cultures showed increased levels of stratification (5–6 cell layers) and differentiation (Figure 4c). The optimum number of days at ALI was determined to be 21 since, after longer times, signs of apoptosis appeared (see Supplementary Figure S2). The presence of an additional 1 mM CaCl_2_ (final calcium concentration 2.25 mM) to the culture medium was fundamental for the 3D growth (for specifications of the ALI culture media, see Supplementary Materials, Table S1). Cultures obtained using the protocol without supplementation of Ca^2+^ (final calcium concentration 1.25 mM) showed reduced cell–cell adhesion and less differentiation after 14 (Figure 4d,e) and 28 days at ALI (Figure 4f). This is likely due to the important role of calcium in cell–cell adhesion (i.e., desmosome formation) and keratinocyte differentiation [24].

However, the degree of stratification and differentiation seen in the split-thickness OME (5–6 cell layers) was not comparable to that observed for the full-thickness OME (7–8 cell layers). It has been shown that the full-thickness oral mucosal equivalent releases more cytokines in comparison to the split-thickness model [25] and that the presence of collagen and fibronectin secretion by fibroblasts in the model play a role in the development of the epithelium [6].

### 3.2. Assessment of Full- and Split-Thickness Oral Mucosa Models for Drug Permeation Studies

One of the most widely accepted quantitative and non-invasive methods to measure the barrier properties of a tissue is the measurement of transepithelial electrical resistance (TEER). In fact, TEER has been proven to measure the integrity of tight junction dynamics in cell culture models of endothelial and epithelial monolayers and the integrity of the superficial cellular layer in tissue-engineered mucosal models [26]. TEER values recorded for the full- and split-thickness models developed here were 118 ± 8 and 140 ± 50 Ω·cm^2^, respectively (Table 1). These values are similar to the 134 ± 28 Ω·cm^2^ recorded for the commercial EpiOral™ model (Table 1) and in agreement with the values reported in the literature for OME [10]. In particular, the in-house-developed OME presented slightly higher TEER values than the 55–122 Ω·cm^2^ reported for SkinEthic-reconstructed human oral epithelial (HOE) model [27].

**Figure 5:**
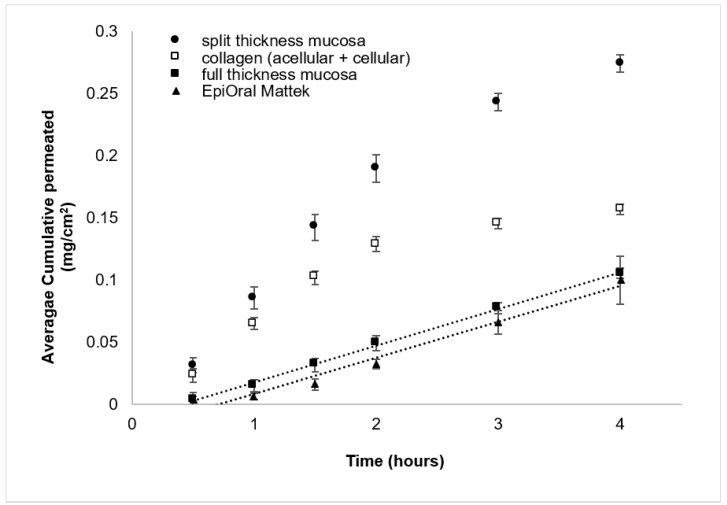
Cumulative amount (mg/cm^2^) of eletriptan hydrobromide (3 mg/mL, pH 6.8) permeated as a function of time. Data are presented as means ± SD (*n* = 4).

The TEER value for EpiOral™ has been previously reported as 413 ± 138 Ω·cm^2^ [28]; however, it is not clear whether this value was obtained after subtracting the value from the empty insert/membrane (i.e., blank), which was accounted for using our method and in the TEER of HOE [27]. TEER values also indicate that split-thickness models, lacking the presence of the lamina propria layer, grow better on polyethylene (PE) or polyethylene tetraphthalate (PET) membranes than on polycarbonate (PC) (Figure S3, Supplementary Materials). However, TEER was not indicative of epithelial stratification since the values registered after 4 days of ALI were similar to the ones after 21 days, while histology images clearly showed differences in stratification at different time points (Figure 4). This might indicate that the epithelial layers in the split-thickness model were not homogeneous, containing patches with weaker barrier properties, which caused a strong reduction in the TEER values of the entire cell-based membrane. To assess the permeation barrier properties of the developed OME models, we performed a drug permeation study using the commercial EpiOral™ model, considered as the reference model for drug permeation and presenting similar epithelial stratification and lipids to the in vivo situation, as a control [7,10]. To investigate the barrier integrity of the full-thickness model, a permeation experiment was conducted on a layer of acellular and cellular collagen (without epithelium). As the model drug for the permeation study, eletriptan hydrobromide (EB) was chosen, a triptan molecule used for the treatment of migraine. For migraine treatment, rapid therapeutic onset is of crucial importance and, therefore, oral transmucosal drug administration could be a promising alternative, reaching higher therapeutic concentrations. It is well known that the hydrophilicity/hydrophobicity balance of the drug drastically affects the drug permeation route. Indeed, we believed that EB was a good model drug since it has a hydrophobic skeleton, but it is charged at physiological pH, being both hydrophobic and hydrophilic at the same time. We recently studied EB permeability in oral transmucosal delivery and the effects of the drug concentration, pH and with a penetration enhancer using model and ex vivo membranes (porcine and 3D commercial OME) [19,29]. The full-thickness model developed here showed the same cumulative drug-permeated profile as the commercial EpiOral™, suggesting that they present the same permeation barrier properties (Figure 5).

This was also confirmed by the similar drug flux values, 28.8 and 29.6 µg/cm^2^/h, for the EpiOral™ and full-thickness model, respectively (Table 2). When the permeation was performed on the lamina propria (i.e., collagen gels: acellular + cellular), the cumulative amount and flux were drastically higher, indicating the relevance of the epithelial layer of the full-thickness model in the permeation barrier. The split-thickness model, which constituted only the epithelial layer, presented a higher drug permeation amount and flux (i.e., 106.3 µg/cm^2^/h), indicating a less tight barrier.

It is difficult to conclude that the barrier property observed in the full-thickness model is due to the addition of the lamina propria plus epithelial barrier, since we have shown previously that the lamina propria has little influence on the EB permeation [19]. Instead, the less tight barrier observed in the split-thickness model is likely due to the reduced stratification in the epithelial layer compared to the full-thickness model (Figure 2 and Figure 3). We have previously linked increased EB permeation at pH 6.8 (i.e., charged form of EB~99%) to the presence of more hydrophilic pathways in the membrane [19], suggesting that our split-thickness model could potentially present more hydrophilic pathways. Although the split-thickness protocol represents a promising, cheaper tissue-engineering protocol, further investigations are needed to reach the same barrier properties as the commercial EpiOral™, considered as the reference model for a drug permeation membrane. Nevertheless, it is not infeasible that the current split-thickness model can be used to study the permeation of other types of drug molecules, such as hydrophobic uncharged drugs. In fact, we have previously shown that the permeation of hydrophobic uncharged drugs (such as EB at pH 10.4) will not be affected if using a membrane presenting a more hydrophilic pathway, since uncharged molecules cannot take advantage of these transport routes [19].

Determining a relationship between permeation barrier properties and TEER values is not trivial. In fact, the TEER values across full- and split-thickness are not significantly different (Table 1). However, permeation data indicate the presence of a more integral barrier for the full-thickness model. After permeation, the TEER value was reduced by 33%, from 118 ± 8 to 79 ± 5 Ω·cm^2^, for the full-thickness model and by 29%, from 140 ± 50 to 97 ± 41 Ω·cm^2^, for the split-thickness model (Table 1). This reduction was found to be significant (*p* < 0.05%) and likely correlated with the fact that the IC_50_ value for EB tested on OKF6 TERT-2 monolayer was found to be 0.745 mg/mL in a previous study [19]. However, after 24 h of recovery in fresh medium, the TEER returned to the initial values, suggesting only a transitional TEER decrease without affecting the tissue integrity. This is in agreement with previous observations that 3 mg/mL EB does not induce toxic effects on 3D OME (i.e., EpiOral™), as seen in histology images [19].

### 3.3. The 3R Approach (Replacement, Reduction and Refinement of Animal-Derived Ingredients) in the Tissue-Engineering Protocol

Tissue-engineering protocols are regulated by a delicate balance between cell proliferation and differentiation, which is modulated by the presence of different ingredients in the growth medium. Among them, fetal bovine serum (FBS) serves most purposes and is the current gold standard to ensure good cell proliferation and differentiation. However, FBS presents significant issues, such as the collection methods and inappropriate cellular growth profiles due to large batch-to-batch variability, as well as possible virus contamination. Replacing FBS is a major challenge since it contains a complex mixture of salts, growth factors, proteins, vitamins, lipids and hormones [30].

**Figure 6:**
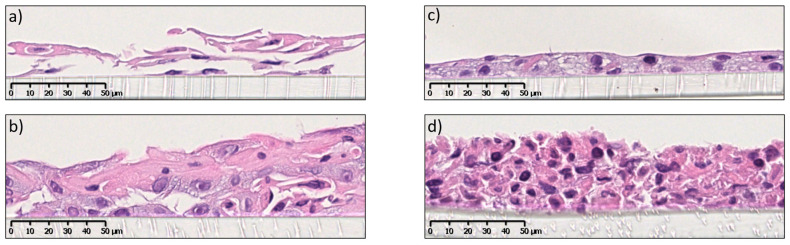
Histological images after H&E staining of split-thickness mucosa cultured in the absence of FBS for (**a**) 15 and (**b**) 28 days; replacing FBS with (**c**) retinoic acid, linoleic acid and EGF and (**d**) retinoic acid, linoleic acid, EGF and BPE. Scale bars are 50 µm.

The split-thickness mucosa cultured in the absence of FBS but in medium supplemented with Ca^2+^ (final calcium concentration 2.25 mM) showed 1–2 flaky layers of undifferentiated cells, confirming that other constituents of the FBS are essential for epithelial stratification (Figure 6a,b). In fact, the switch between proliferation and differentiation is modulated not only by the presence of extracellular calcium but also other factors, such as retinoic acid and growth factors [4]. Epidermal growth factor (EGF, 0.2 ng/mL), together with retinoic acid (10 µM) and linoleic acid (5 µg/mL), was shown to have a positive effect on cell proliferation and differentiation (Figure 6c). Most likely, EGF inhibits apoptosis and promotes basal cell proliferation, as has been observed in the intestinal epithelium [31]. Similarly, the addition of retinoic acid likely regulates differentiation by increasing the number of cell layers in the tissue, in line with previous observations in the oral epithelium [32] and a corneal construct [33]. Linoleic acid has been shown to contribute to the formation of membrane-coating granules [34], compensating for the absence of the lipids from FBS. In fact, although linoleic acid accounts for only 6% of the total free fatty acids in FBS, it is important since a deficiency has been correlated with the incomplete differentiation of murine keratinocytes [35]. Figure 6d shows that bovine pituitary extract (BPE, 25 µg/mL) had a remarkable impact on the number of cell layers, likely due to the promotion of cell proliferation through anti-oxidative effects [36]. However, the obtained OME shows a deficiency in cell–cell contacts and disorganized keratinocyte differentiation.

Even with the addition of the supplements mentioned above to the air-liquid interface medium, some key ingredients appear to be missing that are apparently essential for the full differentiation of the split-thickness model.

### 3.4. Evaluation of the Lipid Composition in Full-Thickness Tissue-Engineered Oral Mucosa

**Figure 7:**
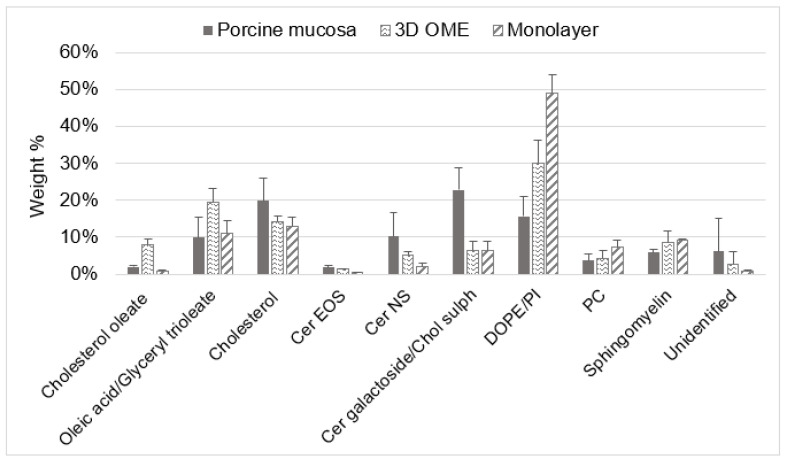
Evaluation of the lipid composition in porcine esophageal mucosa, 2D monolayer (OKF6 TERT-2 keratinocyte) and 3D full-thickness OME. Percentages of ceramide galactoside (Cer galactoside) and cholesterol sulphate (Chol sulph), as well as percentages of oleic acid and glyceryl trioleate, are shown together since the peaks had a minor overlap.

Along with cell proliferation and differentiation, in the non-keratinized epithelial tissue, there is a change in the lipid composition due to the presence of the lipids extruded from the membrane-coating granules (MCG) that originate from the Golgi region of the prickle cell layers and migrate to the upper third layer of the epithelium. These lipids represent the main permeability barrier of the non-keratinized oral mucosa [4]. To understand whether, during the tissue-engineering protocol, there is lipid differentiation, we evaluated the lipid composition of the full-thickness model, which showed a good permeation barrier. As a control, a single-layer epithelial sheet made with OKF6 TERT-2 (i.e., 2D monolayer; see step 2, split-thickness model, Figure 1) was tested to understand the difference in lipid composition during 3D growth, since monolayer cultures are lacking in stratification and intercellular lipids [7]. As a reference model, porcine esophageal mucosa was used since it has been shown previously to present a qualitatively and quantitatively similar lipid composition to that of the human buccal mucosa [37,38,39,40]. We found that in all three samples, the lipids represented less than 10% of the total dry weight (see Supplementary Materials, Table S2). The HPTLC method allowed the separation of all lipid classes on one plate (Figure 7). Reference standard lipids were mixed together and used on each plate to identify and quantify each lipid component. By decreasing the polarity of the mobile phase, phospholipids were resolved first (i.e., sphingomyelin, PC, PI, PE), followed by cholesterol sulphate, ceramide galactoside and ceramides in the middle of the plate. With the apolar solvent mixture in the last few steps of the HPTLC, we resolved cholesterol, oleic acid and glyceryl trioleate and cholesteryl oleate.

Phospholipids (PC, PI and PE) were more abundant in the 2D monolayer, followed by 3D OME and the porcine mucosa, while we observed a general increase in ceramides in the OME compared to the 2D monolayer. The decrease in phospholipids, together with the increase in the ceramide content, is a typical hallmark of the correct lipid differentiation that occurs during epithelium stratification [4,41]. The slight increase in cholesterol from the 2D monolayer (13%) to the 3D OME (14%) and porcine mucosa (20%) corroborates the epithelial differentiation since cholesterol is essential for the spontaneous cross-linked cell envelope formation in terminal differentiation [42]. The high content of fatty acids and related lipids (i.e., glyceryl trioleate and cholesterol oleate) observed for the 3D OME could be due to the presence of fetal bovine serum in the tissue-engineering protocol, as observed previously [41].

The high proportion of ceramide galactoside and the very low abundance of ceramides is in accordance with the nature of non-keratinized epithelia [22]. Ceramide galactoside is transformed into ceramides by glycosidase enzymes in the membrane-coating granules between the intermediate and superficial layers of the stratified epithelium [4]. Regional variation has been observed for the glycosidase activity, which could be the reason for the relatively higher amount of ceramide galactoside in the porcine mucosa that was collected from the lower region of the esophagus [43]. If we compare it with the commercial EpiOral™ MatTek [11], the full-thickness OME developed here presented more ceramide NS (2.4% vs. 5.2%) and long-chain ceramide EOS. Although the ceramide content could be highly implicated in the epithelial barrier function, it did not have a major impact on the permeation of the tested drug molecule, EB (Figure 5).

## 4. Conclusions

To the best of our knowledge, here, we present new insights into both full- (i.e., connective plus epithelial tissue) and split-thickness (i.e., only epithelial tissue) OME using non-tumor-derived human keratinocytes OKF6 TERT-2.

We present a full-thickness model that shows similar barrier properties, transepithelial electrical resistance and lipids to the current benchmark model, EpiOral™. We prove that the model delivers a valid membrane to study the drug permeation of eletriptan hydrobromide, a model drug that presents both hydrophobic and hydrophilic moieties. To corroborate this, lipid analysis on the full-thickness OME revealed a decrease in phospholipids and an increase in ceramides in comparison to the monolayer culture, suggesting that lipid differentiation occurs as a result of the tissue-engineering protocols.

Moreover, we present the first tissue-engineering protocol for split-thickness mucosa using OKF6-TERT-2, the influence of the cell medium composition on the 3D growth and attempts to replace fetal bovine serum. The split-thickness mucosal model was developed directly on porous membranes, and we observed pronounced keratinocyte differentiation resulting in 4–5 cell layers with basal cells still undergoing mitosis. Although the split-thickness protocol is cheaper, the degree of stratification and differentiation was not prominent as in the full-thickness model. Accordingly, the drug flux of EB at pH 6.8 (i.e., charged form of EB~99%) was higher than that observed using the reference model EpiOral™, implying that further investigations are needed to reach the same barrier properties. Following the 3R principles, the addition of Ca^2+^, retinoic acid, linoleic acid, EGF and BPE is important but not sufficient to fully replace fetal bovine serum.

Finally, we believe that the OME models presented here have some advantages since they have a longer shelf-life (at least one week) than the commercial model (2–3 days upon receival), offering broader applications (e.g., longer permeation/irritation studies). Moreover, the model could be used in the future to study the effects of drugs and excipients on keratinocyte differentiation and drug permeation in inflammatory conditions (such as oral lichen planus or xerostomia).

## Figures and Tables

**Table 1:** TEER values across full- and split-thickness OME or the commercial EpiOral™ mucosa model. TEER was measured directly and one week after full ALI differentiation, and directly and after 24 h recovery following drug permeation experiments. Data are presented as means ± SD (*n* = 3). Statistical differences are marked by *: *p* < 0.05 (t-Student).

Sample	TEER Reported (Ω·cm^2^)			
	After ALI Differentiation	1 Week Shelf Life	Directly after Drug Permeation Studies	24 h Recovery after Drug Permeation Studies
Full-thickness mucosa	118 ± 8	112 ± 32	79 ± 5 *	87 ± 43
Split-thickness mucosa	140 ± 50	143 ± 51	97 ± 41 *	110 ± 53
EpiOral™	134 ± 28	24 ± 24	40 ± 40	44 ± 3

**Table 2:** Permeation parameters calculated using eletriptan hydrobromide as a model drug. The flux (*J*) was calculated as the linear regression of the cumulative amount plotted as a function of time, in the time region (0.5–4 h) for the full-thickness oral mucosa and commercial EpiOral™ and (0.5–2 h) for split-thickness oral mucosa and collagen. Data are presented with SD and R^2^ (*n* = 4).

Membrane	*J* (µg/cm^2^/h)	SD (µg/cm^2^/h)	R^2^
EpiOral™	28.8	0.006	0.972
Full-thickness oral mucosa	29.6	0.003	0.998
Split-thickness oral mucosa	106.3	0.106	0.998
Collagen (acellular + cellular)	70.7	0.007	0.991

## Data Availability

Data are contained within the article or supplementary information.

## Supplementary Materials

The following supporting information can be downloaded at: https://www.mdpi.com/article/10.3390/pharmaceutics15051513/s1. Table S1: Specification of the composition of air-liquid-interface medium (ALI) for all tissue engineered mucosa models. Figure S1. Histological image after H&E staining of EpiOral™ Mattek tissue as received and equilibrated according the supplier instructions. Figure S2. Histological image after H&E staining of split-thickness OME cultured for 28 days at the air liquid interface. Figure S3. TEER values for split-thickness mucosa models grown on polycarbonate membrane (PC) or polyethylene derivative insert membranes (PE or PET). Table S2: Specification for the lipid extractions. Figure S4. Electrodes and example of recorded impedance spectrum used in TEER determination.
