# Supplementary material for: Three-Dimensional Oral Mucosal Equivalents as Models for Transmucosal Drug Permeation Studies
Source: Pharmaceutics. 2023 May 17;15(5):1513. doi: 10.3390/pharmaceutics15051513 (PMC10223481; doi:10.3390/pharmaceutics15051513)
Supplement: Supplementary file 1 [file pharmaceutics-15-01513-s001.zip › pharmaceutics-2304105-supplementary.pdf]

Three-Dimensional Oral Mucosal Equivalents as Models for Transmucosal Drug Permeation Studies

Azra Riaz [1,2,†], Sanna Gidvall [1,2,†], Zdenka Prgomet [3], Aura Rocio Hernandez [1,2], Tautgirdas Ruzgas [1,2], Emelie J. Nilsson [1,2], Julia Davies [2,3] and Sabrina Valetti [1,2,*]

[1] Biomedical Science, Faculty of Health and Society, Malmö University, 205 06 Malmö, Sweden
[2] Biofilms–Research Center for Biointerfaces (BRCB), Malmö University, 205 06 Malmö, Sweden
[3] Section for Oral Biology and Pathology, Faculty of Odontology, Malmö University, 205 06 Malmö, Sweden
* Correspondence: sabrina.valetti@mau.se
† These authors contributed equally to this work.

Supplementary information

Contents

Table S1: Specification of the composition of air-liquid-interface medium (ALI) for all tissue engineered mucosa models 2

Figure S1. Histologic images after H&E staining of EpiOral™ Mattek tissue as received and equilibrate according supplier instructions 3

Figure S2. Histologic images after H&E staining of split-thickness OME cultured for 28 days at the air liquid interface 3

Figure S3. TEER values when split-thickness mucosa models were grown on polycarbonate membrane (PC) or polyethylene derivative insert membranes (PE or PET) 4

Table S2: Specification for the lipid extractions 4

Figure S4. Electrodes and example of recorded impedance spectrum used in TEER determination 5

Table S1: Specification of the composition of air-liquid-interface medium (ALI) for all tissue engineered mucosa models

	**Full-thickness mucosa***	**Split-thickness mucosa**				
				**Serum-free medium**		
		**With additional Calcium***	**Without supplement Calcium**	**without FBS**	**without FBS, with retinoic acid, linoleic acid and EGF**	**without FBS, with retinoic acid, linoleic acid EGF and BPE**
**DMEM (4.5g/L)**	x3	x3	x3	x3	x3	x3
**Hams F12**	x1	x1	x1	x1	x1	x1
**$CaCl_2$ (mM)**	1.25 DMEM+ HamsF12	2.25	**1.25**	2.25	2.25	2.25
**FBS %**	5	5	5	**0**	0	0
**Insulin (ug/mL)**	5	5	5	5	5	5
**Hydrocortisone (**µg ml)	0.4	0.4	0.4	0.4	0.4	0.4
**Triiodothyronine (M)**	$2*10^{-11}$	$2*10^{-11}$	$2*10^{-11}$	$2*10^{-11}$	$2*10^{-11}$	$2*10^{-11}$
**Transferrin (µg/ml)**	5	5	5	5	5	5
**Choleratoxin (M)**	10^{-10}	10^{-10}	10^{-10}	10^{-10}	10^{-10}	10^{-10}
**Adenine (M)**	$1.8x10^{-4}$	$1.8x10^{-4}$	$1.8x10^{-4}$	$1.8x10^{-4}$	$1.8x10^{-4}$	$1.8x10^{-4}$
**Glutamine (**mM)	2	2	2	2	2	2
**Penstrep (IU,** ug/ mL)	1	1	1	1	1	1
**Amphotericin B** (ug/ml)	0.5	0.5	0.5	0.5	0.5	0.5
**Retinoic acid (µ**M)	-	-	-		**10**	**10**
**Linoleic acid (µ**g/mL)	-	-	-		**5**	**5**
**EGF**(ng/ml)	-	-	-		**0.2**	**0.2**
**BPE** (ug/ml)	-	-	-	-	-	**25**

*** chosen composition**

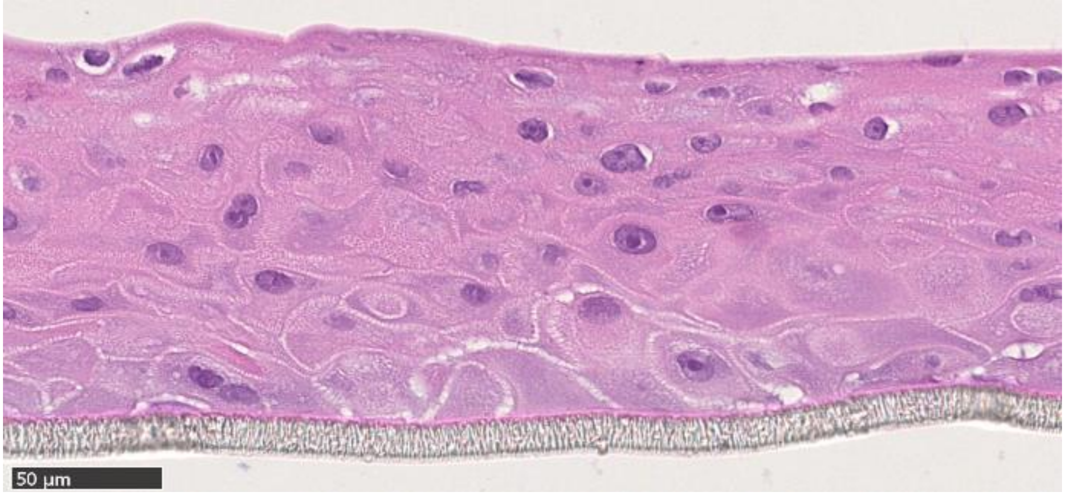

Figure S1. Histological image after H&E staining of EpiOral™ Mattek tissue as received and equilibrated according the supplier instructions.

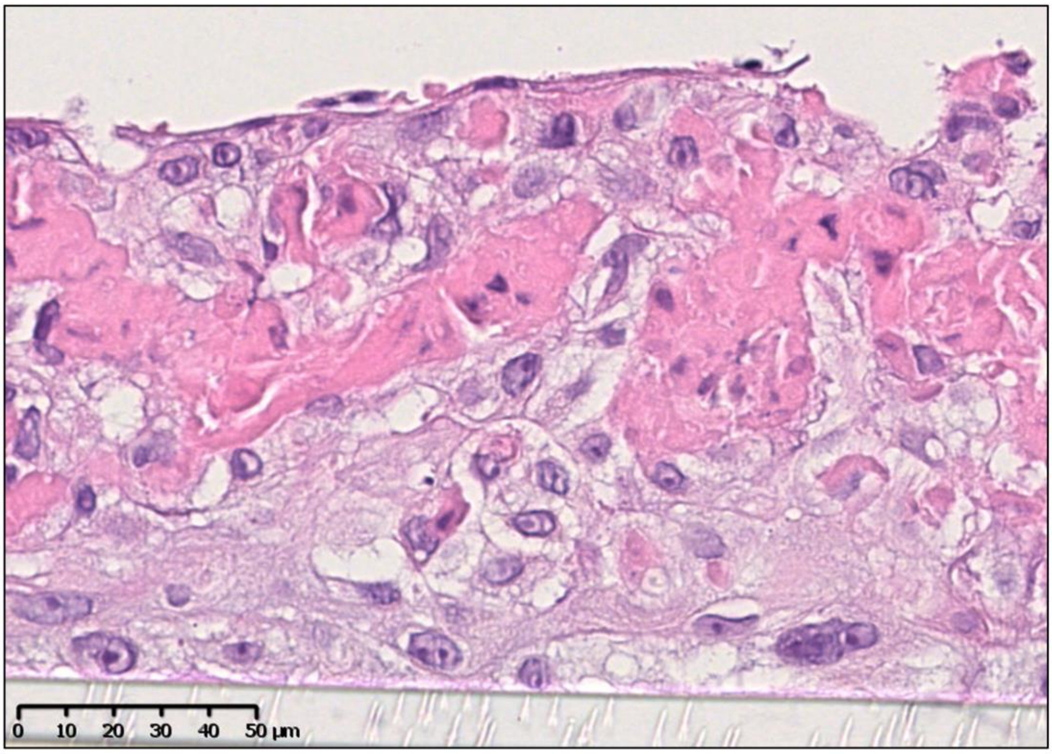

Figure S2. Histological image after H&E staining of split-thickness OME cultured for 28 days at the air liquid interface.

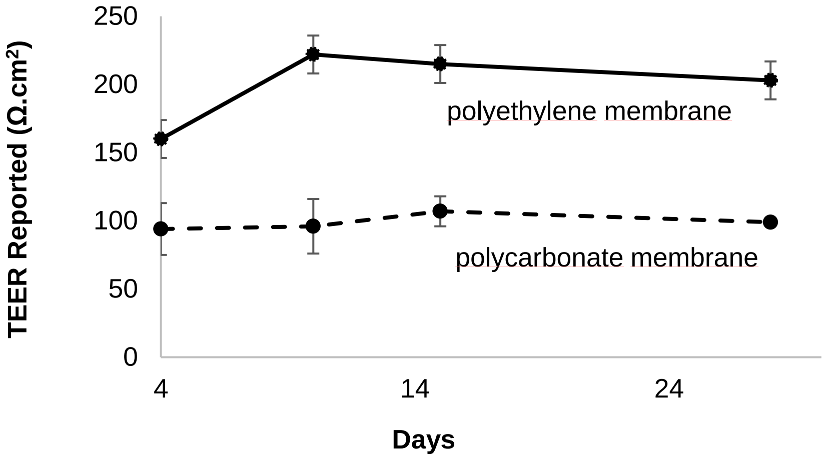

Figure S3. TEER values for split-thickness mucosa models grown on polycarbonate membrane (PC) or polyethylene derivative insert membranes (PE or PET).
Data are presented as means ± SD (n=3).

Table S2: Specification for the lipid extractions

**Tissue**	**Tissue (g)**	**Solvent volume (mL)**	**Extracted lipids (mg)**	**Extracted lipids (dry mass)***
Porcine esophageal mucosa (250 µm thickness)	0.50	500	29	5.8%
Keratinocyte monolayer	0.01	50	1	10%
3D full-thickness OME	0.01	50	<1	<10%

* lipids weight/tissue weight dried

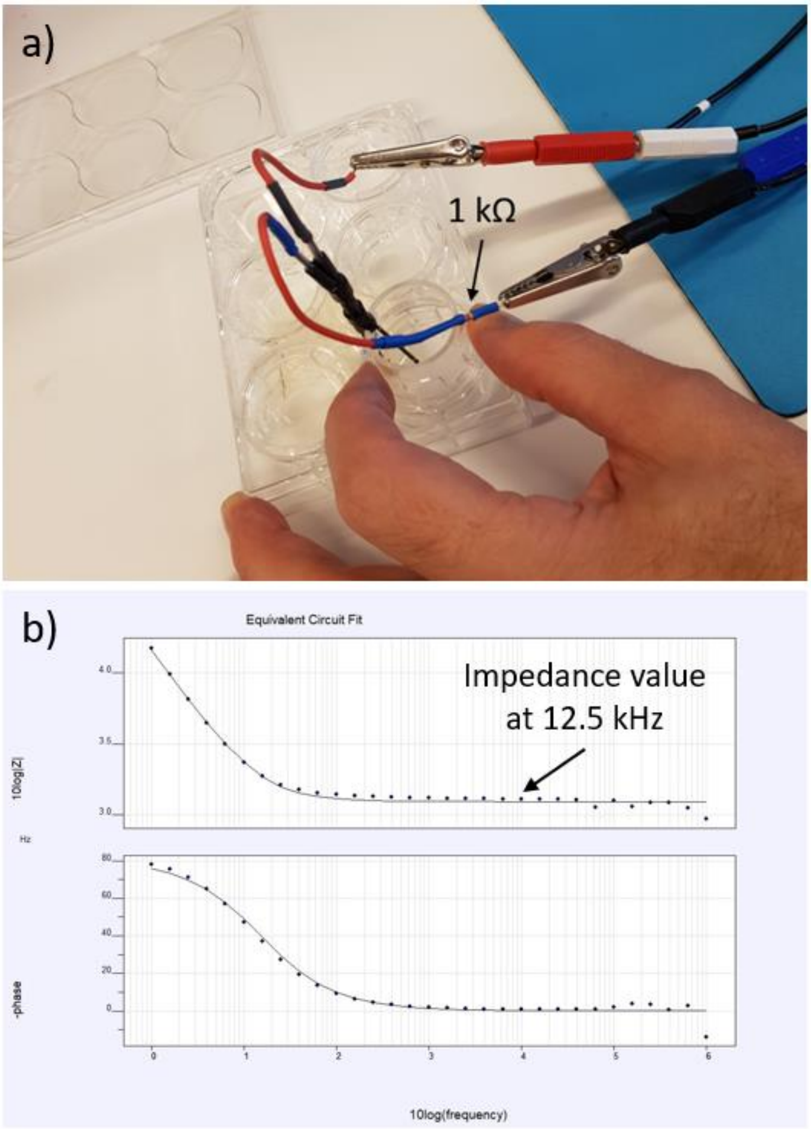

Figure S4. Electrodes and example of recorded impedance spectrum used in TEER determination

(a) Chop-stick electrodes used to determine TEER values of OME. The electrodes were made of two 1 mm diameter glassy carbon rods wound with 0.2 mm diameter Pt wire. The Pt was used to increase the area of the electrodes. The picture shows a connection of 1 kΩ resistor for limiting a current flowing through OME to maximum of 10 μA.

(b) An example of an impedance spectrum recorded for an insert membrane before OME was cultivated. Amplitude of applied AC voltage was 10 mV. The impedance value at 12.5 kHz was used for TEER calculation as described in the main text.
